# Validation of the Clinical Assessment of Prosocial Emotions (CAPE) and Subtype Differentiation of Callous–Unemotional Traits in Children and Adolescents: A Study Protocol

**DOI:** 10.3390/bs16081279

**Published:** 2026-07-27

**Authors:** Felix A. Wolters, Anna Eichler, Oliver Kratz

**Affiliations:** Department of Child and Adolescent Mental Health, Uniklinikum Erlangen, Friedrich-Alexander-Universität Erlangen-Nürnberg (FAU), 91054 Erlangen, Germany; anna.eichler@uk-erlangen.de (A.E.);

**Keywords:** callous–unemotional traits, limited prosocial emotions, disruptive behavior disorders, diagnoses, psychometric properties, clinical assessment of prosocial emotions

## Abstract

Callous–unemotional (CU) traits are associated with specific cognitive and emotional deficits, which help explain their severe aggressive behavior. In the ICD-11/DSM-5, CU traits have been added to the diagnostic criteria for disruptive behavior disorder (DBD) as the “limited prosocial emotions” (LPE) specifier. The semi-structured Clinical Assessment of Prosocial Emotions (CAPE) interview is a new method to identify LPE. This study protocol describes the German translation of the semi-structured CAPE and the planned evaluation of its construct validity in a clinical sample of children diagnosed with DBD in Germany. Given that individuals with elevated CU traits can be categorized into primary (less anxiety, with less experience of abuse) and secondary (high anxiety, with more experience of abuse) variants, this study protocol will outline the planned exploratory analysis of these variants in a German clinical sample. The research questions will be examined in a multicenter, cross-sectional, clinical observational study using a case–control design. The study will include children and adolescents aged 8–18 and their primary caregivers. Multivariable general linear models will be used to assess the construct validity of CAPE and to explore differences between CU trait variants. Validating a German version of CAPE could provide clinicians and researchers with a standardized tool to assess LPE, enabling treatment tailored to CU trait variants. Trial Registration: German Clinical Trials Register, DRKS00035428, registered on 5 November 2024. Ethical approval was obtained from the Friedrich-Alexander Universität Erlangen-Nürnberg (reference number: 24-112-S) on 28 May 2024. Trial status: Recruiting ongoing.

## 1. Introduction

Disruptive behavior disorder (DBD) is a pattern of symptoms that is characterized by oppositional and aggressive behavior that restricts the fundamental rights of others. DBD has been linked to delinquency, substance abuse, criminality, and low educational achievement in children and adolescents (Hyde et al., 2026; Odgers et al., 2007). Given the considerable harm inflicted on society and individuals (Burt et al., 2018), research has identified a high-risk group with callous–unemotional traits (CU traits) and DBD. They are defined by four criteria: a lack of remorse or guilt, a lack of empathy, unconcern about performance, and shallow or deficient affections (Frick et al., 2014).

Children exhibiting CU traits demonstrate substantial impairments in neurocognitive and social–emotional processes, encompassing response inhibition, emotion recognition, response to reward and punishment cues, and affective empathy (Díaz-Vázquez et al., 2024; Fanti et al., 2024; Frick & Kemp, 2021; Hyde et al., 2024; Kimonis et al., 2023; Viding et al., 2012). In earlier studies, children with CU traits showed more persistent and aggressive behavior, had more police contacts over time, showed more antisocial behavior, were more likely to engage in bullying, and proactively used aggressive behavior to achieve their goals compared to unaffected peers (Catone et al., 2021; Frick et al., 2003, 2005; McDonough-Caplan & Beauchaine, 2018; Muñoz & Frick, 2012; Olivera-La Rosa et al., 2023).

Consequently, the presence of CU traits in children with DBD is indicated by the specifier “with limited prosocial emotions” (LPE) in the ICD-11 and DSM-5 (American Psychiatric Association, 2013; Frick et al., 2014). In case of a patient diagnosed with DBD, a specific LPE may be assigned if two CU traits—for example, lack of remorse and guilt, and shallow or deficient affection—persist for longer than one year. Approximately half the children with DBD exhibit elevated levels of CU traits (Kahn et al., 2012; Pisano et al., 2017). In analyses of risk factors for the development of CU traits, research has identified associations between parental characteristics (i.e., low parental warmth, harsh parenting, and parental psychopathology) and an increased likelihood of CU traits in their offspring (Frick & Viding, 2009; Waller et al., 2015, 2017; Wright et al., 2018). Children from families of low socioeconomic status and those from parents with adverse childhood experiences have been linked to higher levels of CU traits, while parental warmth in parenting has been identified as a protective factor (Hyde et al., 2016; Kohlhoff et al., 2020; Pueyo et al., 2022; Tomlinson et al., 2022; Waller et al., 2015).

From a longitudinal perspective, the focus has been on CU traits that develop in response to an earlier traumatic experience (secondary CU traits). A meta-analysis revealed a low to medium correlation between childhood maltreatment experiences and the development of CU traits (Todorov et al., 2023). In this context, CU traits are regarded as survival strategies following traumatic childhood experiences, especially experiences of abuse. The pervasiveness of victimization experiences among individuals characterized by high secondary CU traits may provide a rationale for the consistent lack of moral maturity (Eilts & Bäker, 2024; Kimonis, 2023). This would stand in contrast to primary CU traits, in which traumatic experiences are less prevalent, and deficits in emotion recognition and affective empathy prevail. Studies have shown differences between children and adolescents with primary versus secondary CU traits in aggressive behavior, i.e., proactive aggression in primary CU traits versus reactive aggression in secondary CU traits: In addition, secondary CU traits are associated with elevated scores in CU traits, depression, anxiety, and attention problems (Bennett & Kerig, 2014; Craig et al., 2021; Craig & Moretti, 2019; Dadds et al., 2018; Kahn et al., 2013; Kimonis et al., 2012; Mozley et al., 2018; Vasileva et al., 2019). However, studies on primary and secondary CU traits have rarely addressed traumatic events and have predominantly been based on anxiety intensity (Craig et al., 2021).

### 1.1. Previous Literature and Rationale

To address these open research questions, a valid measurement of CU traits and their subtypes is crucial. CU traits are typically assessed using questionnaires such as the Antisocial Process Screening Device (APSD; (Frick & Hare, 2001)) or the Psychopathy Checklist (PCL-R; (Hare, 2003)). In this context, the Inventory of Callous–Unemotional Traits (ICU (Frick, 2004)) is the current gold standard, characterized by its medium to high test quality (Cardinale & Marsh, 2020) and tripartite subscale structure (callousness, uncaring, and unemotional) (Bhanwer et al., 2019; Fanti et al., 2009; Kimonis et al., 2008, 2015). The merits of the three-factor model structure have also been confirmed in the German version of the child and adolescent self-report (ages 6–18) (Essau et al., 2006). In a clinical German sample, a three-factor model (Callousness/Lack of Guilt, Unemotional, and Unconcerned about Performance) has proven to be the best model fit. However, only Callousness/Lack of Guilt was associated with aggressive/antisocial behavior (Benesch et al., 2014).

Current questionnaires do not facilitate the evaluation of each CU trait individually to diagnose LPE and do not provide clinicians with direct feedback on potential treatment adjustments. This shortcoming contributes to the challenges in developing effective interventions. To address this gap, Frick developed the Clinical Assessment of Prosocial Emotions (CAPE) (Frick, 2013), which is oriented on the DSM-5 criteria of LPE. This semi-structured, thirty-minute clinical interview combines external judgment (from the primary caregiver) with self-judgment (from the child/adolescent). The CAPE was developed to provide a comprehensive assessment of CU traits and to serve as a structured professional judgment (SPJ) tool for diagnosing LPE. Each question is followed by several follow-up questions designed to assess the persistence and frequency of LPE symptoms. The semi-structured CAPE interview requires clinicians to collect specific examples to ascertain whether such behaviors occur across different situations and relationships. This enables us to identify a particularly vulnerable group that requires specialized care.

Within the group of CU traits, a distinction has been demonstrated between two types. However, definitions of primary and secondary CU traits diverge. In particular, the question of the extent to which traumatic experiences (Craig et al., 2021; Kimonis, 2023) and anxiety symptoms (Todorov et al., 2025) contribute to distinguishing the two subtypes is of particular interest. The concept can be traced back to Karpman’s (1941) seminal work, in which he posited that the core of secondary CU traits comprises internalizing symptoms, such as anxiety and depression, in response to a neglectful, unloving, or abusive environment. Porter (1996) expanded on this, referring to severe traumatic experiences in which CU traits are developed as a coping strategy to shut down. The present study protocol will follow this approach and examine both significant potentially traumatic events as well as high anxiety scores in the differentiation process. This is of particular significance given the evidence that children exhibiting secondary CU traits demonstrate a notably diminished response to treatment over time (Fleming et al., 2023). Furthermore, parents encounter a significantly greater challenge in expressing parental warmth (Kaouar et al., 2024) and may necessitate an adapted form of treatment (Kimonis, 2023). In order to achieve this objective, it is necessary to comprehend both primary and secondary CU traits by integrating the two approaches (trauma and anxiety).

### 1.2. Study Aims and Research Questions

Interview formats have proven more effective than current questionnaires for identifying CU traits because the practicing clinician receives direct feedback on which CU characteristics are present. Interviews are the key element for intervention planning. For this objective, the CAPE interview appears to be an appropriate instrument. Unfortunately, neither a German version nor an empirical assessment of the CAPE interview is currently available.

**Research question 1: Can Limited Prosocial Emotions (LPE) be validly diagnosed in a German clinical sample using a German version of CAPE?**

The validity of the CAPE interview data will be examined by correlating it with standardized questionnaire scores on CU traits, DBD, social competence, reactive/proactive aggressive behavior, empathy, emotion recognition, and parental warmth. Furthermore, groups are formed in which children with and without LPE are separated, allowing symptom severity and characteristics to be differentiated. The study at hand will examine the hypothesis that the CAPE is a superior instrument for predicting DBD, social competence, empathy, and emotion recognition compared with questionnaires.

Research has shown that a distinction between primary and secondary CU traits is important. The secondary CU traits result from children’s exposure to one or more traumatic events. This distinction has not previously been examined in a clinical sample from German-speaking countries. This leads to the following exploratory research question:

**Research question 2: Can primary and secondary CU traits be differentiated in a German clinical sample?**

Children diagnosed with DBD and elevated CU traits will be divided into two distinct groups: those with a history of traumatic experiences, i.e., two or more potentially traumatic experiences (secondary), and high anxiety (secondary), compared to those with less and lower anxiety (primary). The objective of this study is to examine the construct of primary and secondary CU traits using standardized procedures. The final two groups will be compared with respect to traumatic events, CU traits, DBD, reactive aggressive behavior, emotion recognition, parental warmth/strictness, attention problems, depression, and anxiety symptoms.

### 1.3. Working Hypotheses

**Construct Validity of CAPE and LPE**

*Interrater reliability and prevalence*

1. The CAPE interviews are expected to demonstrate moderate to very good interrater reliability.
2. It is expected that approximately 50% of children diagnosed with DBD will also exhibit LPE.

*Convergent validity*

3. CAPE will be statistically significantly positively correlated with CU traits, DBD, (proactive) aggressive behavior, and parental strictness.

*Divergent validity*

4. CAPE will be statistically significantly negatively correlated with social competence, empathy, emotion recognition, and parental warmth.

*Incremental validity*

5. The CAPE Interview will demonstrate greater predictive validity than the ICU for DBD, social competence, (proactive) aggressive behavior, empathy, and emotion recognition.

*Group comparison of the specifier “with limited prosocial emotions”*

6. Children with an LPE will differ significantly and positively in CU trait expression, DBD, and (proactive) aggression compared to children without an LPE.
7. Children with an LPE will differ significantly and negatively in social competence, empathy, emotion recognition, and parental warmth from children without an LPE.

**Primary and secondary CU traits**

1. Children with secondary CU traits will show significantly more severe symptoms of depression, attention problems, anxiety, CU traits, reactive aggressive behavior, parental strictness, and DBD than those with primary CU traits.
2. Children with secondary CU traits will be better at recognizing emotions than children with primary CU traits.
3. Children with primary CU traits will have parents who show more parental warmth than those of children with secondary CU traits.

### 1.4. Anticipated Results

Considering the extant research (Colins et al., 2026; Goetz et al., 2024; Hawes et al., 2020; Neo et al., 2023; Molinuevo et al., 2020), it is anticipated that interrater reliability will range within the moderate-to-good range (*κ* = 0.50–0.90). Given that the clinician and the second coder are trained professionals (i.e., psychology master’s candidates, child and adolescent psychotherapists, and/or physicians) who have first observed two sessions to learn how to conduct and evaluate CAPE, it is expected that the study will also demonstrate moderate to high interrater reliability. However, the study’s double-blind design, which relies solely on written notes and visible checked items, with no audio or video recordings, might be a reason that interrater reliability will be significantly lower.

It is currently understood that approximately half of all children diagnosed with conduct disorders have an LPE, in line with previous studies (Colins et al., 2026; Kahn et al., 2013). If this is not the case, it might be attributable to the fact that, although this is a clinical sample with a diagnosed DBD, children exhibiting severe antisocial behavior may be unable to participate due to poor compliance. To understand this, the prevalence of LPE among children in outpatient settings was 21.6% in the most recent CAPE study (Neo et al., 2023). In contrast, in the detention center, it was 60% (Colins et al., 2026), indicating that prevalence can differ between settings.

It is hypothesized that, in terms of convergent validity, CAPE will be positively associated with proactive aggression. We also reckon that children and adolescents with LPE exhibit a higher symptom burden in DBD in both proactive and reactive aggression.

Regarding discriminant validity, it is predicted that patients with LPE will exhibit less developed social competence, empathy, parental warmth, and poorer emotional recognition. In addition, it is anticipated that the discriminant constructs will demonstrate a negative correlation with CAPE.

If the absence of LPE differentiation in proactive aggression and DBD symptom score remains, it may be attributable to aggressive behavior being a core symptom of a diagnosed conduct disorder, which could explain the observed variance. Conversely, LPE might exhibit greater divergence in neurological characteristics, such as emotion recognition (Díaz-Vázquez et al., 2024). Lack of advanced techniques, such as eye tracking, might hinder effective emotion recognition. The fact that we measure empathy globally and do not distinguish between affective and cognitive forms of empathy could explain the lack of a negative correlation (Frick & Kemp, 2021). Furthermore, it is hypothesized that, in the absence of LPE, parents will exhibit greater warmth. There are longitudinal data, particularly extending into middle childhood, that confirm this (Hyde et al., 2016; Tomlinson et al., 2022). However, given such a wide age range, this effect may diminish over time.

Thus, regarding incremental validity, it is hypothesized that CAPE might explain additional variance in the previously referenced convergent and discriminant constructs beyond that explained by the covariates of age and sex, as well as by the ICU. Should this not be the case, it might be attributable to CAPE administration being conducted exclusively with the primary caregiver. Consequently, a review of the child’s records and/or an interview with the child may not capture the full range of variation.

Furthermore, there might be little overall agreement between the primary caregiver’s and the child’s perceptions. This phenomenon may be attributed to the propensity of both children and parents to offer socially desirable responses. Notably, children with conduct disorders exhibit a distinct pattern of social–cognitive information processing and frequently perceive their own behavior as less aggressive (Crick & Dodge, 1994). This factor may also explain a potential lack of clear correlations between the primary caregiver’s rating and the child’s.

The distinction between primary and secondary CU traits is primarily evident in the domains of anxiety and the prevalence of abuse (Cecil et al., 2018; Todorov et al., 2025; Kimonis et al., 2012). Consequently, we assume children and adolescents with secondary CU traits will have higher levels of reactive aggression, poorer attention performance, higher parental strictness, lower parental warmth, higher emotion recognition, and, in general, significantly more pronounced DBD symptoms. This exploratory inquiry aims to determine the extent to which primary and secondary CU traits are also evident in a German clinical sample. The findings may indicate that there are no differences in parental strictness, as strictness may also be considered desirable when it is associated with consistent behavior (Kimonis et al., 2025). Furthermore, factors such as harsh or abusive parental behavior may be central to secondary CU traits, whereas strictness does not reflect this (Facci et al., 2024; Kimonis, 2023).

## 2. Materials and Methods

### 2.1. Setting and Design

This study is a multicenter, cross-sectional, clinical observational study using a case–control design. Data will be collected in outpatient, inpatient, and day-clinic settings of Child and Adolescent Mental Health clinics. The CAPE interview is conducted with the primary caregiver and evaluated by a clinician (LPE, yes/no). Standardized questionnaires are rated by the primary caregiver (external report) and the child (self-report).

### 2.2. Sampling Method, Recruitment, and Procedure

**Figure 1:**
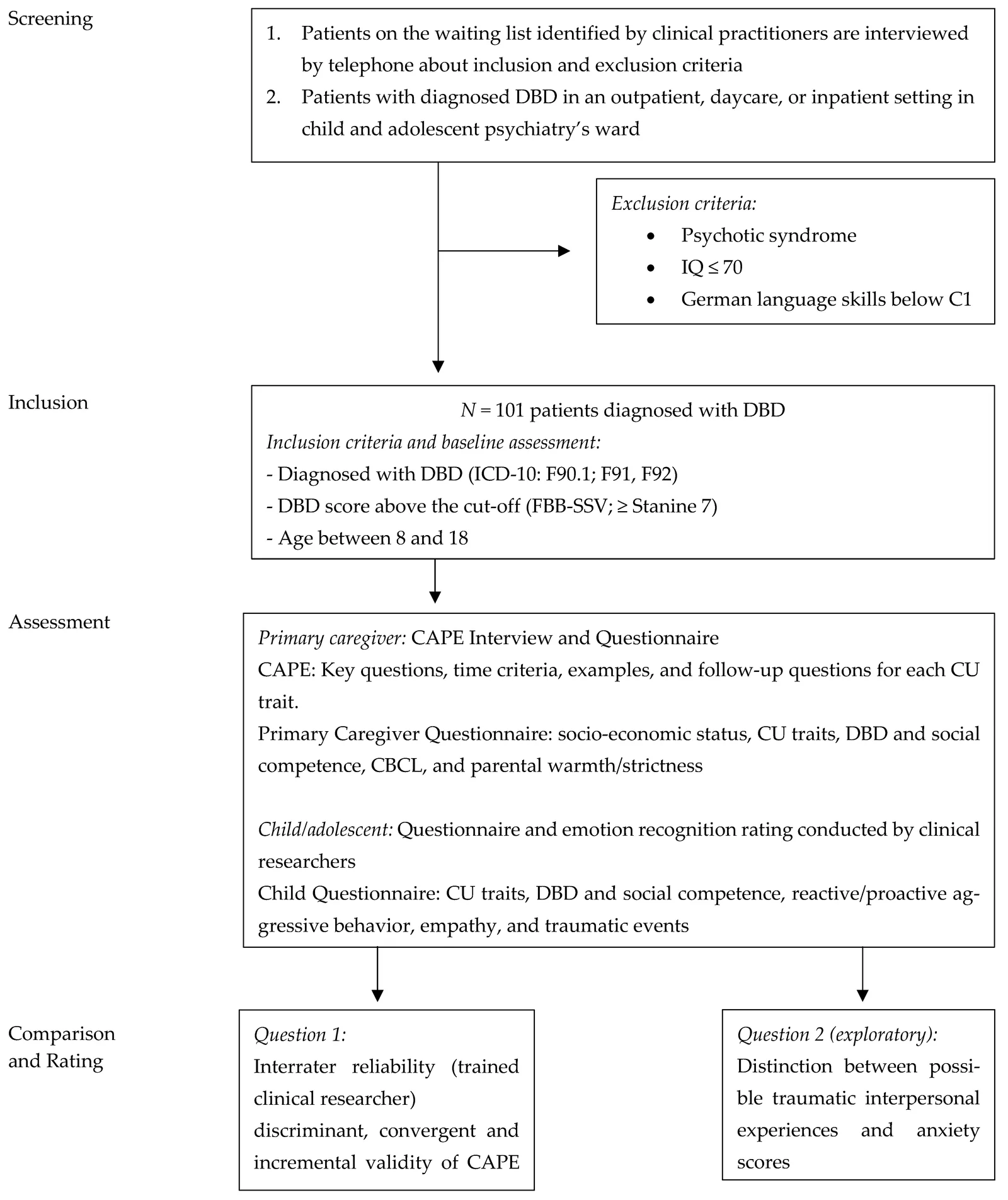
Procedure of the Validation of the Clinical Assessment of Prosocial Emotions (CAPE) and Subtype Differentiation of Callous–Unemotional Traits.

Participants are recruited from two independent clinical sites to improve the generalizability of results and to minimize potential site-specific biases. Parents of children/adolescents undergoing outpatient, inpatient, or day-clinic treatment, or who are on a waiting list for aggressive behavior treatment and diagnosed with DBD, will be contacted by telephone (Figure 1). If interested, study information and consent forms will be sent by post, and an appointment will be scheduled with the primary caregiver and child for data collection. The personal appointment at the Child and Adolescent Mental Health Department will last approximately 1.5 to 2 h. Participants and their primary caregivers are informed about the study, the voluntary nature of participation, data storage, and the procedural aspects involved. After the consent forms are signed, a trained clinician (e.g., a licensed child and adolescent psychotherapist or a trained physician) conducts the CAPE interview with the primary caregiver.

Additionally, the primary caregiver and child complete standardized questionnaires using the electronic data collection software SoSciSurvey^©^ (Version 3.5.07; Leiner, 2024) on a tablet. Due to the study’s format, completion of the study is contingent upon entering all data, thereby ensuring that no entries are missing. As part of the study, the questionnaires are read out to all children. They also have the opportunity to ask questions to make sure they understand. To reduce shared method variance and potential circularity in the validation analyses, SPJ are derived independently from the questionnaires. All families are offered a diagnostic and clarification phase for DBD regardless of their child’s LPE status on the CAPE. Each family is informed that they can share the CAPE results with their psychotherapist or physician.

### 2.3. Inclusion and Exclusion Criteria

Children and adolescents between the ages of 8 and 18 years who exhibit aggressive behavior symptoms above the cut-off (FBB-SSV; (Döpfner et al., 2017): total score ≥ Stanine 7) and who have been diagnosed with DBD (ICD-10: F90.1, F91, F92) will be included. Children and adolescents with acute psychotic symptoms, reduced intelligence (IQ ≤ 70), and a limited understanding of the German language (below C1) will be excluded.

### 2.4. Sample Size Calculation

In line with earlier findings (Colins et al., 2020; Goetz et al., 2024; Hawes et al., 2020; Molinuevo et al., 2020; Neo et al., 2023), we expect medium-to-large effect sizes for research question one when examining and comparing children with and without LPE across relevant convergent/divergent constructs (DBD and social competence, reactive/proactive aggressive behavior, emotion recognition, and empathy). Based on two groups (LPE, yes/no) in an analysis of covariance (ANCOVA), we can estimate, using G*Power 3.1 (effect size = 0.50, α = 0.05, 1−β = 0.95), that a total sample size of 55 would yield statistically significant results. We expect small-to-medium effect sizes when examining LPE correlations with relevant convergent/divergent constructs. Based on calculations (effect size = 0.40, α = 0.05, 1−β = 0.95), we can assume that a sample size of 63 patients would yield statistically significant results. In this context, moderate effects are also assumed when calculating incremental validity via hierarchical regression (effect size = 0.15, α = 0.05, 1−β = 0.95; number of tested predictors: 2; total number of predictors: 7), yielding an *N* of 83.

For research question two, children and adolescents with elevated CU traits will be separated into two groups based on whether they have primary or secondary CU traits, depending on at least two interpersonal potential traumatic events and high anxiety scores. Groups will be compared in levels of anxiety, depression scores, emotion recognition, empathy, DBD, and social competence, and reactive/proactive aggressive behavior (Bennett & Kerig, 2014; Craig et al., 2021; Craig & Moretti, 2019; Dadds et al., 2018; Kahn et al., 2013; Kimonis et al., 2012, 2013; Mozley et al., 2018; Robertson et al., 2023; Vasileva et al., 2019). According to Kimonis and colleagues (Kimonis et al., 2012, 2013), medium-to-high effect sizes are expected (Cohen’s *d* = 0.41–0.96). Kahn and colleagues (Kahn et al., 2012, 2013) reported small-to-medium effect sizes. Hence, we require an *N* of 84 (ANCOVA; G*Power: effect size = 0.40, α = 0.05, 1−β = 0.95) to estimate the required sample size. To adequately address the research question, an *N* of 84 participants is required. Given a 20% attrition rate, this would correspond to 101.

### 2.5. Outcomes

**Clinical Assessment of Prosocial Emotions (CAPE)**

The *Clinical Assessment of Prosocial Emotions* 1.1 (CAPE 1.1 (Frick, 2013)) is a semi-structured interview developed for clinicians to assess CU traits according to DSM-5 criteria in children and adolescents diagnosed with DBD: (a) lack of remorse or guilt, (b) callousness-lack of empathy, (c) unconcerned about performance, and (d) shallow or deficient affect. The interview can be given to the child, parents, teachers, or other caregivers for an SPJ for LPE. In the study at hand, the primary caregiver will be interviewed by a trained and licensed children and adolescent psychotherapist or a trained physician from the Child and Adolescent Mental Health team. We ensure that all CAPE assessments are conducted with legal authorization to perform clinical assessments and with at least three years of experience conducting and evaluating clinical interviews with children and adolescents. These professionals were trained in psychopathology, child development, clinical interviewing, ethical standards, the research literature on CU traits, and the administration and scoring procedures of the CAPE. They are also continuously supervised by the study directors and can ask questions. The interview was translated into German using the TRAPD method (translate, review, adjudication, pretest, documentation) (Curtarelli & van Houten, 2018). During the interview, primary caregivers are asked three yes/no stem questions for each CU trait. In case of a yes response, the interviewer will ask for examples of everyday situations persistent over different settings, (e.g., Does ____ show his/her/their feelings and emotions openly to others? Please give some examples of this), the monthly frequency of the specific behavior, and for how long the symptom has lasted. After the interview, each CU trait is rated by the clinician as ‘not descriptive or mildly descriptive’ (0), ‘moderately descriptive’ (1), or ‘highly descriptive’ (2). If there are two CU traits rated as ‘highly descriptive’, LPE diagnosis is assigned. The CAPE 1.1 assessments in this study were intentionally conducted without using additional sources of information (e.g., files), even though guidelines recommend that interview data should normally be combined with such information (Frick, 2013). In accordance with the methodology by Hawes et al. (2020), the SPJ is administered following the interview with the primary caregiver. This approach is employed to ascertain the interrater reliability of the interview. The necessity for this approach is predicated on the requirement for standardized implementation across multiple study sites.

Furthermore, it reduces the burden on participants and the organizational workload whilst also avoiding overlap in criteria with the child-report questionnaires. For data analysis, the dimensional and dichotomous (LPE yes/no) score will be used. Furthermore, the second, double-blind coder for interrater reliability will be thoroughly prepared to focus on CU traits and their specific characteristics. The secondary coder will have undergone training with one of the original assessors and completed at least two sessions with that assessor before coding CAPEs herself.

Primary caregiver- and child-rated

**CU Traits**: The *Inventory of Callous–Unemotional Traits* (ICU; 11 to 17 years (Frick, 2004)) is a 24-item self-report questionnaire. There are parent- and child-rated versions. A total score can be calculated. Higher scores suggest higher levels of CU traits.

**DBD and social competence:** The *Diagnostic System for Mental Disorders in Children and Adolescents* (DISYPS; 4 to 18 years; (Döpfner et al., 2017)) assesses diagnostic criteria for conduct problems according to ICD-10 and DSM-5 across 55 items. There are parent- and child-rated versions. A total score and six subtest scores (oppositional behavior, aggressive-dissocial behavior, limited prosocial emotions, disruptive affect regulation disorder & irritability, functional impairment, DBD score, and social competence) can be calculated. Higher scores suggest higher symptom levels.

Primary caregiver-rated

**Aggressive behavior, attention problems, physical complaints, depression, and anxiety**: The *Child Behavior Checklist* (*CBCL*; 6 to 18 years (Döpfner et al., 2014)) is a 119-item questionnaire for assessing emotional and behavioral problems in eight primary (anxious/depressed; withdrawn/depressed, physical complaints, social problems, thinking, sleep and repetitive problems, attention problems, rule-breaking behavior, aggressive behavior) and three secondary subscales: internal (anxious/depressed; withdrawn/depressed, physical complaints), external (rule-breaking behavior, aggressive behavior) and general problems (social problems, thinking, sleeping and repetitive problems, attention problems). Higher scores on the scales indicate behavior problems in this area.

**Parental warmth and parental strictness:** The *Parenting Style Inventory* (PSI, no years recommended (Satow, 2013)) assesses parenting behavior using 54 items. A total of six scale values—love, strictness, independence, religiosity, cooperation with a partner, and collaboration with a school—can be calculated. The study will focus on the warmth subscale (10 items; parenting characterized by love, appreciation, and recognition) and the strictness subscale (10 items; parenting characterized by control, rules, and punishment). Higher scores on these scales suggest that the parents are either stricter or warmer.

**Socio-economic status:** Following Lang and colleagues (Lang et al., 2022), a sum index is created based on the educational level of the mother and father (four categories for years in the education system: <9 [1], 9 [2], 10–12 [3] or 13 [4] years), the origin of the mother and father (two categories: international [0] or national [1]) and net monthly family income (six categories: less than 1000 EUR [1], 1000–2000 EUR [2], 2000–3000 EUR [3], 3000–4000 EUR [4], 4000–5000 EUR [5], more than 5000 EUR [6]). There is, therefore, a theoretical range of 3 to 16.

Child-rated

**Proactive aggressive behavior and reactive aggressive behavior**: The *Differential Aggression Questionnaire* (DAF, 10 to 16 years (Petermann & Beckers, 2014)) has three scales (proactive aggression, reactive aggression, and overall aggression) and 16 items. In this study, the scales for proactive and reactive aggression will be used. Higher scores indicate higher levels of aggression.

**Empathy**: The *Questionnaire on Resources in Childhood and Adolescence* (FRKJ; 8 to 16 years (Lohaus & Nussbeck, 2016)) uses 60 items to assess six personal (empathy and perspective-taking, self-efficacy, self-esteem, sense of coherence, optimism, self-control) and six social (parental emotional and social support, authoritative parenting style, peer group integration, school integration) resources of children and adolescents. Only the empathy subscale (6 items) is used in this study. Higher scores suggest higher levels of empathy.

**Emotion recognition**: The *Diagnostic and Therapeutic Procedure for Accessing Emotions in Children and Adolescents* (EMO-KJ, 5–16 years; (Kupper & Rohrmann, 2018)) offers a child-rated questionnaire (23 items) for emotion recognition (happy, sad, angry, anxious, shy, disgusted, proud, and ashamed). The emotion recognition scores are binary (yes/no). In this study, a sum score is used as an emotion recognition score.

**Traumatic event:** The *Children and Adolescent Trauma Screen 2* (CATS-2; 7–17 years (Sachser et al., 2022)) is a questionnaire used to assess criteria for post-traumatic stress disorder according to DSM-5. On the first page, 14 potentially traumatic events are recorded as yes (1) or no (0); on the second, clinical symptoms are rated from never (0) to almost always (3). In this study, the total number of affirmed potentially traumatic events is calculated, with a possible range from 0 to 14.

### 2.6. Statistical Analysis

All constructs are procedures that have been validated in Germany; the manualized procedure was adhered to. It is important to note that all scales, except those designed for DBD and social competence, are expressed as total scores. DBD and social competence were utilized as means.

**Research question 1:**

*Interrater reliability*

To ascertain interrater reliability, all CAPE interviews will be independently coded by a second rater in a double-blind (no information on questionnaire data and SPJ from the treating clinician) coding process. The secondary coder will either be a psychology student (master’s candidate), a trained child and adolescent psychotherapist, or a trained physician. We will use quadratically weighted Cohen’s κ to estimate interrater reliability.

*Convergent and divergent validity*

Pearson correlations are used to assess discriminative validity (empathy, emotion recognition, parental warmth, social competence) and convergent validity (proactive aggression, DBD, CU traits, aggressive behavior, parental strictness).

*Incremental validity*

Hierarchical regression analyses will be conducted to determine whether the dimensional CAPE score can serve as an incremental predictor of DBD, social competence, proactive aggressive behavior, empathy, emotion recognition, and the control variables of age and gender beyond the ICU.

*Group comparison of the specifier “with limited prosocial emotions”*

This study aims to investigate the hypothesis that children with or without CAPE LPE differ in levels of CU traits, DBD, and social competence, parental warmth/strictness, (reactive/proactive) aggressive behavior, empathy, and emotion recognition. To this end, an analysis of covariance (ANCOVA) will be performed. We will control for age, study site (waiting list, outpatient, daycare, or inpatient), traumatic event, anxiety (to account for primary and secondary CU traits), and sex in the ANCOVA models.

**For research question 2:**

*Defining groups*

Patients with ‘highly descriptive’ in at least one CU Trait on the CAPE interview will be separated into two groups (primary or secondary CU traits), theory-informed by the experience of two interpersonal potentially traumatic events (≥2 = secondary CU traits) and by anxiety scores (T ≥ 70 = secondary CU traits). This approach requires that the SPJ assessment include at least one CU characteristic, thereby ensuring that the exploratory subgrouping is limited to patients who exhibit clinically relevant CU-related characteristics. It is imperative to acknowledge that this exploratory classification does not aspire to represent or supersede a formal LPE classification. We concur with Cecil et al. (2018) that data-driven methodologies, such as cluster analysis, are not feasible in a clinical context, where decisions are made according to cutoff points. Moreover, previous analyses have demonstrated that trauma allows for a distinction between primary and secondary CU traits (Cecil et al., 2018; Kimonis et al., 2012), and there is also evidence that anxiety effectively differentiates between the two forms (Todorov et al., 2025). Consequently, we adhere to the recommendations set forth by Craig et al. (2021) and undertake a comprehensive assessment of potentially traumatic experiences and anxiety levels. In this study, a cutoff of two potentially traumatic experiences will be used to account for severity and to model repeated interpersonal trauma (Karpman, 1941; Kimonis et al., 2012; Pueyo et al., 2022).

*Group comparison*

ANCOVA models assessing group differences between primary and secondary CU trait groups across relevant constructs (CU traits, emotion recognition, reactive aggressive behavior, depression, attention problems, anxiety, parental warmth/strictness, and DBD) will be conducted. We will control for age, study site (waiting list, outpatient, daycare, or inpatient), and sex in the ANCOVA models.

*Effect size and statistical assumption*

In accordance with the study by Neo et al. (2023), the *κ* values will be interpreted using Altman’s (1991) guidelines: values ranging from 0.80 to 1.00 will be classified as indicating very good agreement, values ranging from 0.60 to 0.80 will be classified as indicating good agreement, values ranging from 0.40 to 0.60 will be classified as indicating moderate agreement, values ranging from 0.20 to 0.40 will be classified as indicating fair agreement, and values below 0.20 will be classified as indicating poor agreement. Effect sizes will be estimated using η^2^ (small = 0.01, medium = 0.06, and large = 0.14 (Cohen, 1988)). To correct for multiple testing, Alpha levels will be Bonferroni-Holm corrected.

Before conducting the ANCOVA, a comprehensive series of prerequisite checks will be conducted. These checks encompass the identification of potential outliers (±3 standard deviations), the assessment of linearity between covariate and dependent variables through scatter plots, the evaluation of homoscedasticity via residual plots, and the confirmation of the normal distribution of the disturbance variables through histograms of the standardized residuals and P-P plots. Independence of observations will be addressed through the study design. Multicollinearity is evaluated using variance inflation factors (VIFs). The assumption of homogeneity of regression slopes will be tested by examining the interaction between the grouping variable and the covariate.

## 3. Ethics and Trial Registration

The Ethics Committee of the Medical Faculty at Friedrich-Alexander Universität Erlangen-Nürnberg approved the project (committee reference number: 24-112-S) on 28 May 2024. In addition, the project was registered in the German clinical trials registry (ID: DRKS00035428).

Participants will be informed about the study procedure, its benefits, and associated risks. Before the study begins and interviews are recorded, written informed consent will be obtained from parents and the child’s legal guardians. Participation in the survey can be withdrawn at any time without giving reasons. All procedures follow the principles of the Declaration of Helsinki, 1964, and Good Clinical Practice.

## 4. Discussion

Currently, the only method of assessing CU traits in German-speaking countries is through questionnaires. In a German clinical sample, the ICU shows mixed results regarding discriminant and convergent validity, as well as the factor structure (Benesch et al., 2014). Furthermore, stanine scores can be generated using the LPE subscale of the DISYPS III (Döpfner et al., 2017). None of the questionnaires capture the full concept of each of the four CU traits individually, nor can they accurately measure LPE. Validating CAPE for LPE would provide clinicians with an additional tool for diagnosing CU traits. This is important with respect to the LPE specification in the newly published ICD-11 and the adaptation of treatment (Fanti et al., 2024; Frick & Viding, 2009).

Thus, due to the deficits mentioned in the processing of fearful, neutral, and angry faces in patients with high CU traits (De Brito et al., 2025), as well as the associated neurological characteristics—such as reduced neural processing of others’ pain perception and amygdala hypoactivity (Hyde et al., 2024) — those affected are limited in their ability to recognize and process emotions and to build (affective) empathy. Furthermore, their reactions to time-outs or negative consequences are limited (Kimonis et al., 2025). Thus, the treatment implications suggest that they could benefit from a tailored approach (Hyde et al., 2026), especially when parents are involved from an early age (Perlstein et al., 2023; Waller et al., 2017).

Recent studies using CAPE and DSM-5 LPE criteria have produced mixed findings. While LPE has been linked to higher CU traits and lower empathy (Hawes et al., 2020; Molinuevo et al., 2020), group differences in behavioral problem severity have been inconsistent, likely due to the use of small, heterogeneous samples and binary measures of LPE. On the other hand, recent studies (Goetz et al., 2024; Neo et al., 2023) provided more substantial support for the dimensional validity of the CAPE, demonstrating that children with LPE had higher CU traits, more externalizing problems, lower empathy, and worse treatment outcomes. These findings might support the use of a dimensional, developmentally sensitive assessment of LPE, which we aim to validate in our German sample.

Furthermore, the findings on parental warmth were evaluated in only one study using CAPE (Colins et al., 2026), and it appears that not all studies included participants with a diagnosed DBD alone (Goetz et al., 2024; Hawes et al., 2020; Molinuevo et al., 2020; Neo et al., 2023). Unlike previous studies, this study aims to provide a more accurate picture of clinical reality because it only includes patients who have been diagnosed with DBD. This means that CAPE is being tested under more realistic conditions. Parental training programs are considered essential for treating CU traits (Perlstein et al., 2023), and parental warmth is a key factor in their development (Hyde et al., 2016; Tomlinson et al., 2022). In this study, parental warmth can be considered to identify initial associations with specific traits and to assess how the maintenance components of DBD and LPE could be addressed more specifically.

The study at hand broadens the array of constructs compared to those employed in previous research: in addition to the relevant CU trait constructs (Frick et al., 2018), it encompasses empathy, emotion recognition, proactive aggressive behavior, and parental warmth. Although the CAPE interview is conducted with the primary caregiver only, CAPE scores are based not on raw caregiver ratings but on clinician SPJ derived from a semi-structured interview. This distinguishes the CAPE from questionnaire-based parent reports and represents a distinct assessment modality in which trained raters evaluate LPE criteria using structured probing and contextualized information. At the same time, the interview’s informational basis is limited to a single primary informant.

An additional strength of this approach is that CAPE ratings are based on caregiver interviews, without access to caregiver- or child-reported questionnaire data. This reduces shared method variance and potential confirmation bias while still allowing independent cross-informant validation using questionnaire measures from both sources. Future studies may benefit from incorporating additional informants’ perspectives directly into the interview process to further examine the role of informant-specific information in assessing CU traits.

This study hopes to benefit from considering a broader range of potential risk factors, extending beyond the anxiety scores previously examined by Craig and colleagues (Craig et al., 2021). Previous studies have generally used cluster analysis and latent profile analysis to distinguish primary from secondary CU traits (Craig & Moretti, 2019; Kimonis et al., 2012), but few have focused on clinical cutoffs. Data-driven approaches (e.g., cluster analysis) are often impractical in clinical settings because treatment and risk decisions typically rely on thresholds. Although continuous trait modeling can capture the full range of scores and clarify the links between CU traits and anxiety, they are difficult to implement in clinical practice (Cecil et al., 2018). This study offers an opportunity to explore depression, aggression, and attention using cut-off values to determine whether primary and secondary CU traits can be identified. Combining an anxiety cut-off, as recommended by Todorov et al. (2025) based on a large multinational dataset, with a history of maltreatment, as defined by Craig et al. (2021), allows us to investigate the potential usefulness of this approach in identifying CU trait subtypes in patients diagnosed with DBD. Another salient point is that the nature of the potentially traumatic event might influence the development of secondary CU traits. For instance, trauma occurring within a family context may have a greater impact on the development of secondary CU traits than other forms of interpersonal trauma (Craig et al., 2021). This should be taken into account in future (longitudinal) analyses.

Although the local study protocol for validating CAPE controls for sex, sex-specific analyses for children with LPE have not been explicitly addressed. This does not negate the importance of considering them. Evidence suggests that boys exhibit more pronounced CU traits (Falcón et al., 2021; Moore et al., 2017) and that CU traits tend to emerge earlier in boys during puberty and later in girls (Pueyo et al., 2024). Moreover, evidence indicates that neuroanatomical changes in the brain (particularly in the amygdala) occur specifically in boys (Ibrahim et al., 2021). Consequently, future studies should incorporate sex-specific analyses, including those tailored to the novel LPE.

Furthermore, genetic aspects have not yet been sufficiently accounted for in the new specification, as approximately half of the CU traits point to genetic factors (Takahashi et al., 2021). In contrast, environmental influences can mitigate these traits in an age-specific manner (Waller et al., 2017). Consequently, future studies are most likely to incorporate genetic aspects with greater rigor.

## 5. Limitations

A limitation of this study is that the SPJ is based solely on the primary caregiver’s information from the semi-structured interview. On the other hand, this approach provides a consistent source of information, particularly regarding reliability across age groups, and prevents potential overlap with the child-reported questionnaire measures used to assess convergent and discriminant validity, which could otherwise inflate correlations. The current design allows a more rigorous assessment of construct validity by keeping interview-based ratings separate from self-reported questionnaire data. At the same time, including both caregiver- and child-reported questionnaire measures is hoped to improve the investigation of cross-informant relationships, and to offer a more comprehensive evaluation of the CAPE. This method will have allowed trained clinical raters to evaluate LPE criteria using standardized probing and clinical judgment while maintaining a consistent informational basis across participants. Given this limitation, it is important to determine whether, beyond cross-confirmation validity, discrepancy criteria and social desirability controls should be implemented in future analyses. Limitations yet to be considered include limited ecological validity, the absence of a teacher’s perspective, the lack of systematic integration of case records, and potential caregiver bias. Consequently, only a proportion of CAPE’s potential applications will be statistically and clinically evaluated.

A further limitation might be that primary and secondary CU traits are distinguished solely by a trauma-and-anxiety cutoff, and no separate cluster analysis or other advanced statistical methods will have been used, as has been the case in previous research (Kimonis et al., 2012; Todorov et al., 2025). However, it should be noted that the concept of primary and secondary CU traits encompasses much more than just exposure to traumatic events and high levels of anxiety. It also includes affective emotional functioning and high physiological arousal (Porter, 1996), harsh parenting, and significant parental psychopathology (Kimonis, 2023), as well as associated high scores on internalizing disorders (Cecil et al., 2018), which are only partially reflected here.

A longitudinal study is considered worthwhile, particularly to assess the prognostic validity of CAPE and to examine the continued bidirectional effects of trauma and anxiety on secondary CU traits after two years. Given the current cross-sectional design, no causal conclusions can be drawn. Despite the study’s multicenter design, the number of sites is limited, which restricts the sample’s representativeness and, consequently, the generalizability of the results.

## 6. Conclusions

This study protocol outlines the validation of a German version of CAPE in a multicenter clinical sample and documents standardized procedures for deriving LPE ratings. The assessment, based on the study protocol, may strengthen the evaluation of callous–unemotional traits and support future research on primary and secondary CU traits. It may demonstrate how the SPJ, following the CAPE interview with the primary caregiver, can be incorporated into the treatment plan in a more time-efficient manner. Identification of LPE might help clinicians customize treatment, especially by improving emotion recognition (Fanti et al., 2024) and by targeting parental interventions, including positive reinforcement and parental warmth (Bjørnebekk & Mørkrid Thøgersen, 2022; Fleming et al., 2022; Perlstein et al., 2023).

Furthermore, distinguishing between primary and secondary CU traits using cutoffs (trauma and anxiety) might allow for a more accurate representation of clinical reality and enable examination of initial correlations in a German clinical sample. Given the absence of interventions specifically designed to target secondary CU traits (Kimonis, 2023) and the evidence that secondary CU traits are associated with poorer treatment outcomes over time (Fleming et al., 2023), a comparison of the core characteristics of social and emotional deficits in CU traits within the context of this study offers a valuable opportunity to enhance our understanding of secondary CU traits. This enhanced understanding, in turn, can inform the development of an appropriate treatment approach.

Therefore, accurate diagnosis and treatment adaptation for patients with CU traits are essential for the success of any psychotherapy, especially when they can prevent adverse long-term outcomes and provide significant social benefits by reducing violence and crime over time (Frick et al., 2005, 2014, 2018; Frick & Viding, 2009; Frick & White, 2008; Muñoz & Frick, 2012).

## Data Availability

The datasets used and/or analyzed during the current study are available from the corresponding author on reasonable request.

## Abbreviations

CAPE	Clinical Assessment of Prosocial Emotions
CATS-2	Children and Adolescent Trauma Screen 2
CBCL	Child Behavior Checklist
CU	Callous–Unemotional
DAF	Differential Aggression Questionnaire
DBD	Disruptive behavior disorder
DISYPS	Diagnostic System for Mental Disorders in Children and Adolescents
DSM	Diagnostic and Statistical Manual of Mental Disorders
EMO-KJ	Diagnostic and Therapeutic Procedure for Accessing Emotions in Children and Adolescents
FRKJ	Questionnaire on Resources in Childhood and Adolescence
ICD	International Statistical Classification of Diseases and Related Health Problems
ICU	Inventory of Callous–Unemotional Traits
LPE	Limited prosocial emotions
PSI	Parenting Style Inventory
SPJ	structured professional judgment
